# Effects of IL-6 on pyruvate dehydrogenase regulation in mouse skeletal muscle

**DOI:** 10.1007/s00424-013-1399-5

**Published:** 2013-11-14

**Authors:** Rasmus S. Biensø, Jakob G. Knudsen, Nina Brandt, Per A. Pedersen, Henriette Pilegaard

**Affiliations:** 1 Centre of Inflammation and Metabolism and The August Krogh Centre, University of Copenhagen, Copenhagen, Denmark; 2 Department of Biology, University of Copenhagen, Copenhagen, Denmark; 3 Present Address: Exercise and Metabolic Disease Research Laboratory, Translational Sciences Section, School of Nursing, University of California, Los Angeles, CA USA; 4 Universitesparken 13, 2100 Copenhagen Ø, Denmark

**Keywords:** IL-6, Pyruvate dehydrogenase activity, AMPK, p38, Skeletal muscle

## Abstract

Skeletal muscle regulates substrate choice according to demand and availability and pyruvate dehydrogenase (PDH) is central in this regulation. Circulating interleukin (IL)-6 increases during exercise and IL-6 has been suggested to increase whole body fat oxidation. Furthermore, IL-6 has been reported to increase AMP-activated protein kinase (AMPK) phosphorylation and AMPK suggested to regulate PDHa activity. Together, this suggests that IL-6 may be involved in regulating PDH. The aim of this study was to investigate the effect of a single injection of IL-6 on PDH regulation in skeletal muscle in fed and fasted mice. Fed and 16–18 h fasted mice were injected with either 3 ng · g^−1^ recombinant mouse IL-6 or PBS as control. Fasting markedly reduced plasma glucose, muscle glycogen, muscle PDHa activity, as well as increased PDK4 mRNA and protein content in skeletal muscle. IL-6 injection did not affect plasma glucose or muscle glycogen, but increased AMPK and ACC phosphorylation and tended to decrease p38 protein content in skeletal muscle in fasted mice. In addition IL-6 injection reduced PDHa activity in fed mice and increased PDHa activity in fasted mice without significant changes in PDH-E1α phosphorylation or PDP1 and PDK4 mRNA and protein content. The present findings suggest that IL-6 contributes to regulating the PDHa activity and hence carbohydrate oxidation, but the metabolic state of the muscle seems to determine the outcome of this regulation. In addition, AMPK and p38 may contribute to the IL-6-mediated PDH regulation in the fasted state.

## Introduction

Skeletal muscle has an exceptional ability to adjust the substrate choice during fasting and exercise according to the substrate availability [9, 24]. During prolonged exercise, skeletal muscle first oxidizes blood glucose and stored glycogen, but an increasing fraction of the energy is derived from fat oxidation as the exercise proceeds [37, 38]. This ensures that exercise can continue, although at a lower exercise intensity. Similarly, fasting is associated with a switch in substrate oxidation from carbohydrates to fat markedly increasing survival without food intake.

The pyruvate dehydrogenase complex (PDC) is central in the regulation of substrate choice in skeletal muscle. PDC converts pyruvate into acetyl CoA thereby linking glycolysis with the citric acid cycle and represents the only entry for carbohydrate-derived substrate into the mitochondria for oxidation. PDC is composed of several copies of three catalytic enzymes, which includes the pyruvate dehydrogenase (PDH)-E1α. Fasting has been shown to downregulate the activity of PDH in the active form (PDHa) [43]. In addition, PDHa activity increases with exercise [37], but declines towards resting level as the exercise duration exceeds 2 h [25, 39]. Although PDHa activity can be allosterically regulated [2], phosphorylation/dephosphorylation of the α subunit of PDH-E1 is thought to be the main mechanism regulating the activity of PDH. Phosphorylation and concomitant inactivation of PDH is catalyzed by pyruvate dehydrogenase kinases (PDK), and dephosphorylation and concomitant activation of PDH is catalyzed by pyruvate dehydrogenase phosphatases (PDP). The decrease in PDHa activity during fasting seems to be due to increased PDK4 protein and decreased PDP1 protein content in the muscle [43], while the reduction during prolonged exercise does not appear to be caused by increased PDK4 protein content but may be due to increased PDK activity as previously reported [39]. The underlying mechanisms initiating the regulation of PDH during prolonged exercise remains unresolved.

Interleukin (IL)-6 is a myokine released from contracting muscle during exercise and suggested to exert autocrine, paracrine and endocrine effects [23]. The plasma IL-6 concentration has been reported to increase in both humans and mice during prolonged exercise [20, 21]. In humans, the increase in plasma IL-6 concentration during exercise is detectable after about 2 hour with moderate exercise intensity and increases further with exercise duration [12, 34], while an increase in plasma IL-6 has been detected already after 30 min of intense exercise in mice [20]. Recombinant IL-6 has been shown to increase free fatty acid release from myotubes [1] and increase fat oxidation in human skeletal muscle in vivo [35, 42]. In addition, IL-6 has been reported to increase AMP-activated protein kinase (AMPK) phosphorylation in rat skeletal muscle [13], and rodent studies suggest that AMPK may regulate PDH [17, 32]. Furthermore, IL-6 has been shown to increase phosphorylation of the stress mitogen activated protein (MAP) kinase p38 in incubated human muscle [6] and MAPK p38 has been demonstrated to be involved in the regulation of IL-6 transcription [36]. Furthermore, previous findings suggest an association between increased p38 phosphorylation and decreased PDHa activity in cardiomyocytes [29]. Together this suggests that IL-6 could be involved in regulating the substrate choice of muscle either by directly regulating PDH or potentially via AMPK and p38. Therefore, the aim of the present study was to examine the isolated effects of a single injection of recombinant mouse (rm) IL-6 on PDH regulation in mouse skeletal muscle. As IL-6 normally is elevated during exercise in the presence of severe metabolic challenge, the effect of IL-6 injection was determined in both the fed and fasted state.

## Methods

### Mice

Female C57BL/6 mice (Taconic, Lille Skensved, Denmark) 8 weeks of age were used in the study. Female mice were used because a previous study showed no indications of gender-specific regulation of PDH [14] and because regular oestrous cycle has been reported to be absent in the majority of female mice housed in large groups [41]. The mice had ad libitum access to water and normal chow diet (Altromin 1324, Brogaarden, Lynge, Denmark) and had a 12:12 light–dark cycle. The experiments were approved by the Danish Animal Experimental expectorate (license no. 2009, 561 1607) and complied by the European Convention for the protection of vertebrate animals used for experiments and other scientific purpose (Council of Europe no. 123. Strasbourg, France 1985).

### Experimental setup

The mice were housed individually 24 h before initiation of the experiment and divided into two groups either fed or fasted for 16–18 h. The mice were given an intraperitoneal injection of either phosphate-buffered saline (PBS) or rm IL-6 (3 ng · g^−1^) dissolved in PBS. The mice were euthanized by cervical dislocation 30 or 60 min after the injection. Quadriceps muscles were removed and frozen in liquid nitrogen. Trunk blood was collected in EDTA containing tubes and plasma was collected after centrifugation at 2,600×*g*, 15 min, 4 °C. Muscle and plasma samples were stored at −80 °C.

### Plasma glucose and plasma IL-6

Plasma glucose concentration was analyzed fluorometrically [18]. The plasma IL-6 concentration was determined using Meso Scale Discovery (Rockville, MD, USA) as described by the manufacturer.

### Muscle glycogen

Muscle samples were hydrolyzed in 1 M HCl and the muscle glycogen concentration was determined fluorometrically as glycosyl units as previously described [22].

### RNA isolation and reverse transcription

Quadriceps muscles from the mice were crushed in liquid nitrogen to ensure homogeneity of each sample. RNA was isolated using a guanidinium thiocyanate phenol-choloroform method as previously described [4, 26] except that the samples were homogenized using a Tissue LyserII (Qiagen, Hilden, Germany). Reverse transcription was performed using the Superscript II RNase H^-^ system (Invitrogen, Carlsbad, CA, USA) as previously described [26].

### Real time PCR

mRNA content was determined by use of the 5′ fluorogenic nuclease assay with TaqMan probes (ABI PRISM 7900 Sequence Detection System; Applied Biosystems, Foster City, CA, USA) as previously described [19]. Primer and probe sequences are given in Table 1. Cycle threshold values reflecting the content of a specific mRNA were converted to an arbitrary amount by use of a standard curve, constructed from a serial dilution of a representative sample run together with the unknown samples. For each sample the amount of target cDNA was normalized to the total single stranded (ss)DNA content in the sample determined by OliGreen as previously described [19].

**Table 1:** Primer and Taqman probes

	Forward primers	Reverse primers	Taqman probes
IL-6	5′GCTTAATTACACATGTTCTCTGGGAAA3′	5′CAAGTGCATCATCGTTGTTCATAC3′	5′ATCAGAATTGCCATTGCACAACTCTTTTCTCAT′3
PDK4	5′GGAAGTATCGACCCAAACTGTGA′3	5′GGTCGCAGAGCATCTTTGC3′	5′CACTCAAAGGCATCTTGGACTACTGCTACCA3′
PDP1	5′CGGGCACTGCTACCTATCCTT′3	5′ACAATTTGGACGCCTCCTTACT3′	5′AGTGGCACAAGCACCCCAATGATTACTTC3′

Sequences of forward and reverse primers as well as Taqman probes used for real-time PCR

*IL-6* interleukin 6, *PDK4* pyruvate dehydrogenase kinase 4, *PDP1* pyruvate dehydrogenase phosphatase 1

### SDS-PAGE and western blotting

Crushed quadriceps muscles were homogenized using a Tissue LyserII (Quagen, Hilden, Germany) and muscle lysate was prepared as previously described [25]. The protein content in each sample was determined using the bicinchoninic acid method (Pierce, Rockford, IL, USA).

Protein content and protein phosphorylation were determined in muscle lysates using pre casted gels (Biorad, Hercules, CA, USA) or hand-casted gels and SDS-PAGE and Western blotting as previously described [16, 25] with primary polyclonal antibodies against Signal Transducer and Activator of Transcription (STAT)3 (9139; Cell Signaling Technology, Berverly, MA, USA), p38 (9212; Cell Signaling Technology), acetyl CoA carboxylase (ACC; P0397; DAKO, Glostrup, Denmark), AMPKα2, PDP1, PDK4, and PDH-E1α (provided by Professor Graham Hardie, Dundee University, Dundee, UK) and primary phospho-specific antibodies against STAT3 Tyr^705^ and AMPK Thr^172^ (9138 and 2535, respectively, Cell Signaling Technology), ACC Ser^79^ (07-303; Millipore, Bedford, USA), PDH-E1α site 1 (Ser^293^), site 2 (Ser^300^), and site 4 (Ser^295^; provided by Professor Graham Hardie). Secondary antibodies used were all species-specific horseradish peroxidase conjugated immunoglobulin (DakoCytomation, Glostrup, Denmark). The bands were visualized with ECL reagent (Millipore, Billerica, MA, USA) in a photo image system and quantified (Carestream Health, Rochester, NY, USA). The PDP1 protein band was verified with recombinant protein. GAPDH protein was determined (2118, Cell Signaling Technology) and showed no changes between groups.

### PDHa activity

PDHa activity was determined in muscle homogenates as previously described [3, 5, 25, 31]. PDHa activity in each sample was adjusted to the total creatine content in the homogenate as previously described [33].

### Statistics

Values are presented as means ± SE. Two-way ANOVA tests were used to evaluate the effect of fasting and IL-6 injection by testing within each time point and within each condition. The data were log transformed if equal variance test failed. If a main effect was observed, the Student–Newman–Keuls post hoc test was used to locate differences. In addition, a Student’s *t* test was used to evaluate the effect of IL-6 within a given time point and condition. Differences were considered significant when *P* < 0.05 and a tendency is reported for 0.05 ≤ *P* < 0.1. The statistical tests were performed using SigmaPlot 11.0.

## Results

### Plasma IL-6

**Fig. 1:**
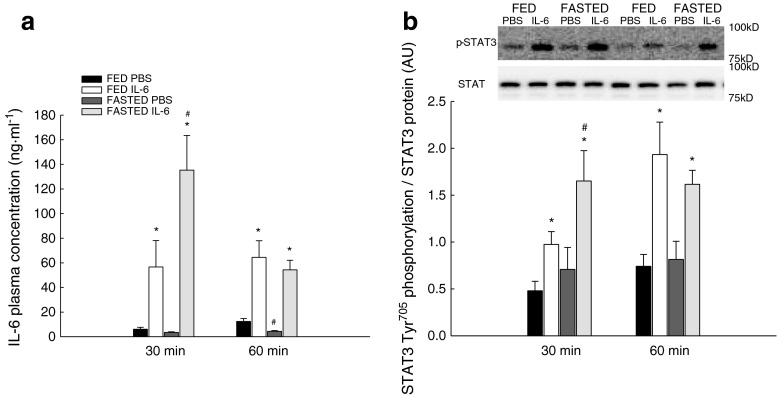
Plasma concentration of IL-6 and STAT3 phosphorylation. Plasma IL-6 concentration (nanograms per milliliter) (**a**) and STAT3 phosphorylation (**b**) in fed (*FED*) and fasted (*FASTED*) mice 30 and 60 min after a single injection of either PBS or rmIL-6. Values are means ± SE; *n* = 8. *P < 0.05, significantly different from PBS within given condition and time point; #*P* < 0.05, significantly different from FED within given treatment and time point

The plasma IL-6 concentration was measured to examine the time course of plasma IL-6 after an IL-6 injection. The IL-6 injection increased (*P* < 0.05) the plasma IL-6 concentration 9.5-fold in fed mice and 40-fold in fasted mice 30 min after the injection relative to the PBS injected mice. After 60 min, the plasma IL-6 concentration was still elevated (*P* < 0.05) 5-fold in fed mice and 12-fold in fasted mice relative to the PBS-injected mice (Fig. 1a).

In the IL-6-injected mice, the plasma IL-6 level was 2.4-fold higher (*P* < 0.05) in fasted than fed mice at 30 min, which may be due to differences in blood volume or differences in uptake or removal of IL-6 from the blood. In the PBS-injected mice, fasting reduced (*P* < 0.05) the plasma IL-6 concentration to 60 % compared with the fed mice (Fig. 1a). However, of notice is that such effect was not present at 30 min suggesting that additional factors have contributed to the effect observed at 60 min (Fig. 1a).

### STAT3 phosphorylation

STAT3 phosphorylation was determined in skeletal muscle to confirm that IL-6 injections induced IL-6-mediated intracellular responses.

IL-6 injection increased (*P* < 0.05) STAT3 Tyr^705^ phosphorylation in skeletal muscle 2-fold in fed mice and 2.3-fold in fasted mice relative to PBS at 30 min after injection. At 60 min, the injection of IL-6 increased (*P* < 0.05) the STAT Tyr^705^ phosphorylation in skeletal muscle 2.2-fold in fed and 2-fold in fasted mice relative to PBS-injected mice (Fig. 1b).

In the IL-6-injected mice, STAT3 Tyr^705^ phosphorylation was at 30 min 1.8-fold higher (*P* < 0.05) in the fasted than the fed mice (Fig. 1b).

### Plasma glucose

**Fig. 2:**
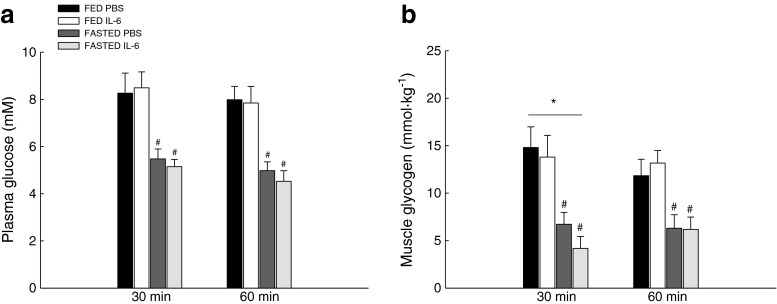
Plasma glucose concentration and muscle glycogen. Plasma glucose concentration (millimolar) (**a**) and muscle glycogen (millimoles per kilogram) (**b**), in skeletal muscle of fed (*FED*) and fasted (*FASTED*) mice 30 and 60 min after a single injection of either PBS or rmIL-6. Values are means ± SE; *n* = 8. *P < 0.05, significantly different from PBS within given condition and time point; #*P* < 0.05, significantly different from FED within given treatment and time point. *Line* indicates an overall effect

The blood glucose concentration was measured to describe the metabolic status of the mice during fasting and after IL-6 injection. The IL-6 injection did not change the plasma glucose concentration relative to PBS, neither in the fed nor the fasted mice (Fig. 2a).

Plasma glucose concentration was at approximately 8 mM in fed mice independent of injection and decreased (*P* < 0.05) to approximately 5 mM in the fasted mice both in the PBS and the IL-6-injected mice (Fig. 2a).

### Muscle glycogen

Muscle glycogen concentration was measured to describe the metabolic status of the mice during fasting and after IL-6 injection. There was a main effect (*P* < 0.05) of IL-6 injection on muscle glycogen content at 30 min, but no changes were observed at 60 min (Fig. 2b).

Fasting reduced (*P* < 0.05) the muscle glycogen content with 50–60 % compared with the fed mice independent of injection (Fig. 2b).

### Signaling

AMPK, ACC and p38 phosphorylation were determined to examine the effects of IL-6 on intracellular signaling in skeletal muscle.

#### AMPK

**Fig. 3:**
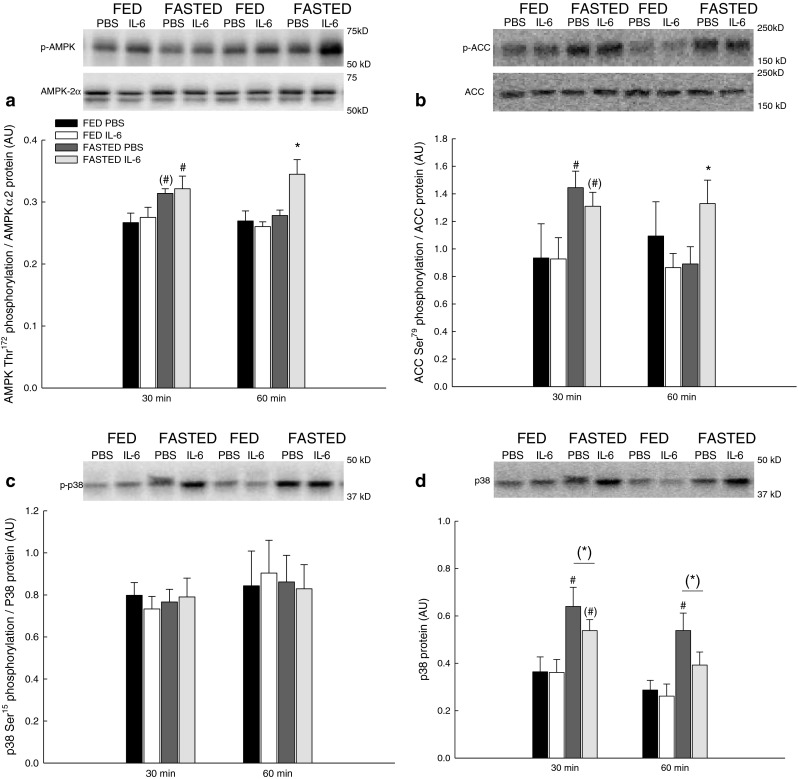
Phosphorylation of AMPK, ACC, and p38. AMPK Thr^172^ phosphorylation (**a**), ACC Ser^79^ phosphorylation (**b**), p38 Ser^15^ phosphorylation (**c**), and p38 protein content (**d**) in skeletal muscle of fed (*FED*) and fasted (*FASTED*) mice 30 and 60 min after a single injection of either PBS or rmIL-6. Values are means ± SE; *n* = 8. *P < 0.05, significantly different from PBS within given condition and time point; #*P* < 0.05, significantly different from FED within given treatment and time point. *Symbols within parentheses* indicate a statistical tendency, 0.05 ≤ *P* < 0.1. *Line* indicates an overall effect

There were no effects of IL-6 on AMPK Thr^172^ phosphorylation in skeletal muscle of the fed mice. In the fasted mice, the IL-6 injection increased (*P* < 0.05) AMPK Thr^172^ phosphorylation 1.3-fold relative to PBS at 60 min (Fig. 3a).

In addition, at 30 min fasting tended to increase (0.05 ≤ *P* < 0.1) AMPK Thr^172^ phosphorylation 1.2-fold in the PBS-injected mice and in the IL-6-injected mice fasting increased (*P* < 0.05) AMPK phosphorylation 1.2-fold relative to fed (Fig. 3a).

#### ACC

The IL-6 injection did not change ACC Ser^79^ phosphorylation in skeletal muscle of the fed mice. In the fasted mice, the IL-6 injection increased (*P* < 0.05) the ACC phosphorylation 1.4-fold in the fasted mice relative to the PBS mice at 60 min when using a *t* test (Fig. 3b).

In line with the AMPK results, 30 min after injection, ACC Ser^79^ phosphorylation was 1.5-fold higher (*P* < 0.05) in fasted than in fed mice injected with PBS and tended to be 1.4-fold higher (0.05 ≤ *P* < 0.1) in fasted than in fed mice injected with IL-6 (Fig. 3b).

#### p38

The IL-6 injection did not change the phosphorylation level of p38 when normalized to p38 protein content in skeletal muscle of fed or fasted mice (Fig. 3c). However, the IL-6 injection tended to decrease (0.05 ≤ *P* < 0.1) the p38 protein content with 70–85 % compared with PBS injection both at 30 and 60 min (Fig. 3c, d).

In addition, p38 protein content was 1.5- to 1.8-fold higher (*P* < 0.05) in the fasted than the fed mice both at 30 and 60 min (Fig. 3d).

### IL-6 mRNA

**Fig. 4:**
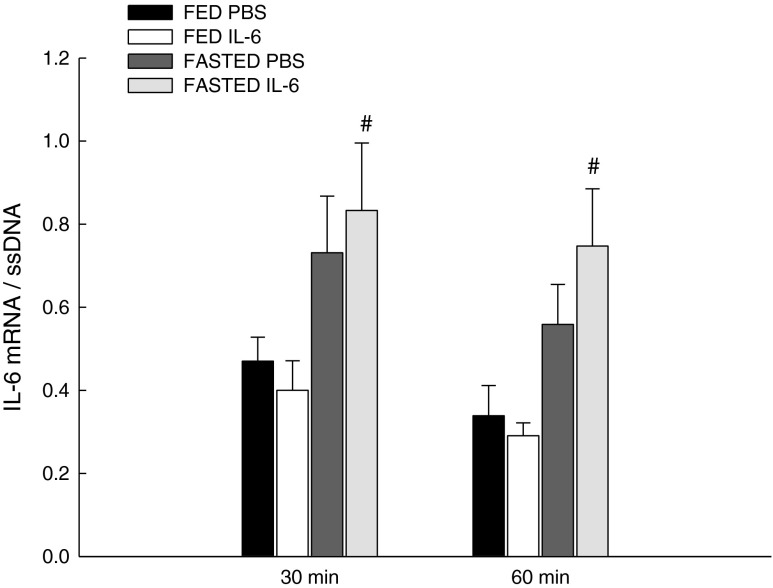
IL-6 mRNA content. IL-6 mRNA in skeletal muscle of fed (*FED*) and fasted (*FASTED*) mice 30 and 60 min after a single injection of either PBS or rmIL-6. Values are means ± SE; *n* = 8. #*P* < 0.05, significantly different from FED within given treatment and time point

IL-6 injection did not change the mRNA content of IL-6 in skeletal muscle of the fed and fasted mice (Fig. 4).

Furthermore, fasting increased (*P* < 0.05) the IL-6 mRNA level 1.8- to 2.0-fold at 30 and 60 min but only in the IL-6-injected mice (Fig. 4).

### PDH regulation

The mRNA and protein levels of PDK4 and PDP1 were determined together with the PDHa activity and PDH phosphorylation to examine the effects of fasting and IL-6 injection on PDH regulation.

#### PDK4 and PDP1 mRNA

**Fig. 5:**
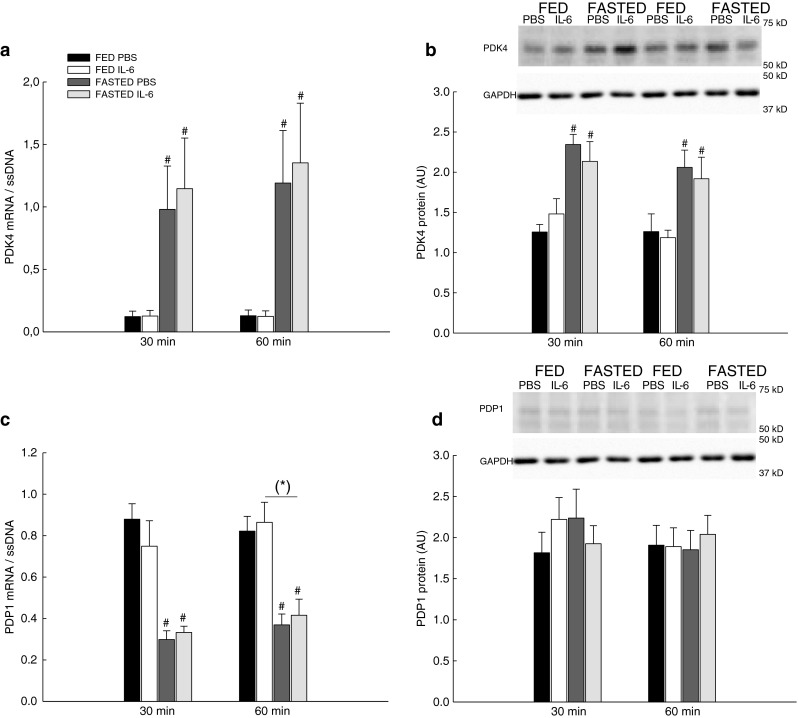
mRNA and protein content of PDK4 and PDP1. PDK4 mRNA (**a**), PDK4 protein content (**b**), PDP1 mRNA (**c**), and PDP1 protein content (**d**) in skeletal muscle of fed (*FED*) and fasted (FASTED) mice 30 and 60 min after a single injection of either PBS or rmIL-6. Values are means ± SE; *n* = 8. *P < 0.05, significantly different from PBS within given condition and time point; #*P* < 0.05, significantly different from FED within given treatment and time point. *Symbols within parentheses* indicate a statistical tendency, 0.05 ≤ *P* < 0.1. *Line* indicates an overall effect

IL-6 injection did not change the PDK4 mRNA content in skeletal muscle of the fed and fasted mice (Fig. 5a), but the PDP1 mRNA content tended to be higher (0.05 ≤ *P* < 0.1) 60 min after IL-6 injection than PBS (Fig. 5c).

Fasting increased (*P* < 0.05) the PDK4 mRNA content 5- to 6-fold Fig. 5a) and decreased (*P* < 0.05) the PDP1 mRNA content with 60 % (Fig. 5c).

#### PDK4 and PDP1 protein

The PDK4 protein content in skeletal muscle increased (*P* < 0.05) 1.6-fold in the fasted mice relative to the fed with no effect of IL-6 injection (Fig. 5b). The PDP1 protein content did not change with IL-6 injection or with fasting (Fig. 5d).

#### PDHa activity

The PDHa activity in skeletal muscle tended overall to be lower (0.05 ≤ *P* < 0.1) in the IL-6-injected fed mice than the PBS injected fed mice. In addition, the PDHa activity was in the fed mice at 30 min 30 % lower (*P* < 0.05) after IL-6 injection than after PBS when using a *t* test. IL-6 injection in the fasted mice increased (*P* < 0.05) PDHa activity 2-fold at 30 min and 1.8-fold at 60 min.

**Fig. 6:**
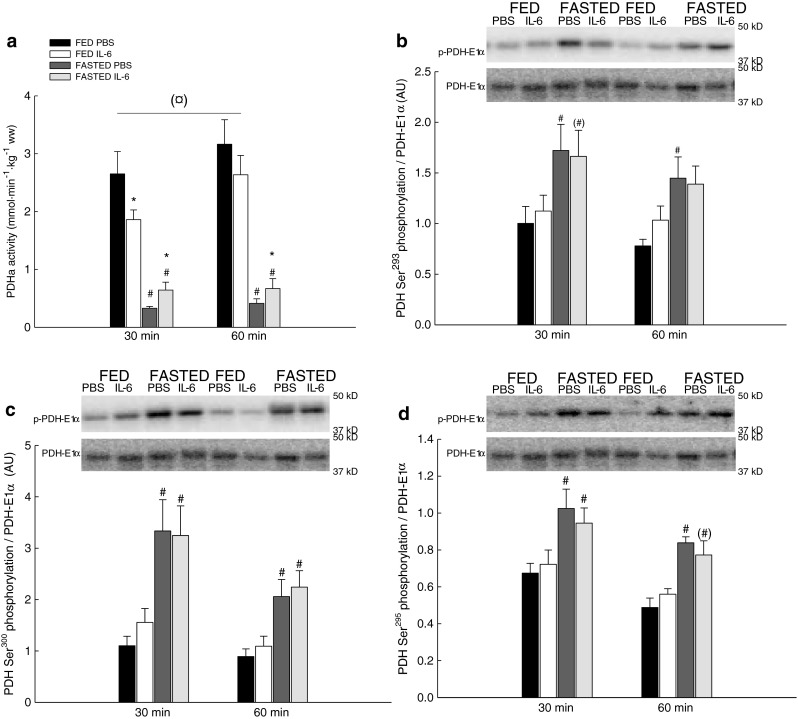
PDHa activity and phosphorylation of PDH. PDHa activity (millimoles per minute per kilogram) (**a**), PDH Ser^293^ phosphorylation (**b**), PDH Ser^300^ phosphorylation (**c**), and PDH Ser^295^ phosphorylation (**d**) in skeletal muscle of fed (*FED*) and fasted (*FASTED*) mice 30 and 60 min after a single injection of either PBS or rmIL-6. Values are means ± SE; *n* = 8. *P < 0.05, significantly different from PBS within given condition and time point; #*P* < 0.05, significantly different from FED within given treatment and time point; ¤0.05 ≤ *P* < 0.1, tendency for a difference between IL-6 and PBS. *Symbols within parentheses* indicate a statistical tendency 0.05 ≤ *P* < 0.1. *Line* indicates an overall effect

Furthermore, fasting overall reduced (*P* < 0.05) the PDHa activity in skeletal muscle to 15–30 % of the level in fed mice (Fig. 6a).

#### PDH-E1α phosphorylation

Overall, the effects on PDH-E1α phosphorylation were similar for the investigated PDH-E1α phosphorylation sites. IL-6 injection did not change PDH-E1α phosphorylation in the fed or fasted mice (Fig. 6b–d).

Fasted mice had 1.7- to 2.1-fold higher (*P* < 0.05) PDH-E1α phosphorylation than fed mice independent of injection (Fig. 6b–d).

## Discussion

The main finding of the present study is that IL-6 injections regulated PDHa activity in mouse skeletal muscle but differently in fed and fasted conditions. In addition, an IL-6 injection increased AMPK and ACC phosphorylation in mouse skeletal muscle only in the fasted state. This indicates that AMPK did not mediate the IL-6-induced reduction in PDHa activity in the fed state but could potentially be involved in the increase in the fasted state. Furthermore, IL-6 reduced p38 protein content in skeletal muscle in the fasted state, making it possible that p38 could play a role in the observed IL-6-induced change in PDHa activity in the fasted state.

Skeletal muscle regulates substrate choice according to availability and is therefore capable of coping with major metabolic changes during exercise and fasting [2, 38]. The previous findings that IL-6 infusion in humans enhances fat oxidation in skeletal muscle [35] and that plasma IL-6 is increased during prolonged exercise [21] suggest that IL-6 could be involved in regulating substrate choice during exercise. The observation that the increase in plasma IL-6 after the IL-6 injection in the present study was similar to the increase reported after a single exercise bout in mice [20], shows that the obtained IL-6 levels are at a physiologically relevant level. Furthermore, the increase in phosphorylation of the IL-6 signaling marker STAT3 in skeletal muscle in the current study demonstrates that IL-6 elicited an intracellular response in skeletal muscle at the investigated time points. Together this suggests that the present experimental setting can be used as a model to investigate the isolated effects of IL-6 on PDH regulation in skeletal muscle during exercise. In addition, the pronounced fasting-induced decrease in muscle glycogen and plasma glucose concentration shows that the fasted mice were severely metabolically challenged. This provides a model to investigate the potential impact of the metabolic state for IL-6-mediated effects on PDH regulation in skeletal muscle.

The observation that IL-6 downregulated PDHa activity in skeletal muscle in the fed state is in accordance with the previously shown IL-6-induced fat oxidation [35, 42] and suggests that IL-6 may contribute to regulating substrate choice during exercise towards increased skeletal muscle fat oxidation in part by regulating PDH. The previous observation that an IL-6 injection increased AMPK phosphorylation in rat skeletal muscle suggests that AMPK may be mediating IL-6-induced regulation [13]. Furthermore, AMPKα2 knock-out (KO) mice have been reported to exhibit an enhanced exercised-induced increase in PDHa activity [17] and the AMPK activator AICAR has been shown to increase PDK4 mRNA in skeletal muscle [11]. Together this suggests that IL-6 mediates effects on PDH via AMPK. However, the present observation that AMPK and ACC phosphorylations were unaffected by IL-6 injections in the fed state does not support that AMPK was directly involved in the observed IL-6-induced reduction in PDHa activity in the fed state.

The present findings that IL-6 elicited an increase in skeletal muscle PDHa activity in the fasted state, despite the IL-6-induced downregulation in the fed state, indicate that the metabolic status influences the impact of IL-6 on PDH regulation. As IL-6 injections in the fasted state were associated with increased AMPK and ACC phosphorylation at 60 min, it is possible that AMPK was involved in the IL-6-mediated PDH regulation at this time point. However, this potential effect of AMPK on PDH is opposite of the apparent AMPK-mediated suppression of the exercise-induced PDHa activation, as suggested from the more marked exercise-induced increase in PDHa activity in AMPKα2 KO than wild-type (WT) mice [17]. However, the AMPKα2 KO mice in the previous study had lower muscle glycogen and plasma glucose after exercise than WT [17]. Therefore, the more marked PDH activation in AMPKα2 KO mice may not be due to the lack of AMPK, but rather that the AMPKα2 KO mice were more metabolically challenged than WT as also suggested in the previous study [17, 25]. Furthermore, as low muscle glycogen has been suggested to enhance exercise-induced IL-6 expression and release from skeletal muscle [12] the more marked PDHa activation in the AMPKα2 KO mice in the previous study [17] may have been due to increased IL-6 levels rather than lack of AMPK. This possibility is supported by the previous finding that AICAR incubation elicited a higher IL-6 release from AMPK kinase dead mouse muscle than WT muscle [7] and is also in line with the present IL-6-mediated increase in PDHa activity in the fasted state. In addition, the present findings that IL-6 injection tended to reduce p38 protein content in the fasted state may suggest that p38 potentially have contributed to the observed IL-6-induced increase in PDHa activity, because increased p38 phosphorylation has been associated with decreased PDHa activity in cardiomyocytes [29]. Of notice, the IL-6-induced downregulation of p38 protein only 30 and 60 min after injection is surprisingly fast and indicates that IL-6 either inhibits p38 synthesis or increases degradation. While a previous study has shown that IL-6 regulates p38 phosphorylation in skeletal muscle [40], no previous studies have to our knowledge reported IL-6-mediated regulation of p38 protein. However, it may be speculated that IL-6 mediates the regulation of p38 mRNA targeting miRNA’s leading to reduced translation of p38, but this remains to be determined.

As PDHa activity is known to be regulated by changes in NADH/NAD+, ATP/ADP, and acetyl CoA/acetyl [9, 24, 30], it may be speculated that IL-6 has mediated effects via changes in one of these ratios. Indeed a previous study has shown that IL-6 incubation of rat EDL decreased the ATP concentration and elevated the AMP concentration suggesting that IL-6-induced changes in nucleotides could play a role in the observed IL-6-mediated regulation of PDHa activity in the present study. An IL-6-induced decrease in ATP and increase in AMP concentrations would be expected to be associated with an enhanced PDHa activity, which is in accordance with the changes observed in the fasted state in the present study. However, changes in ATP, ADP and AMP as well as NADH/NAD+ and acetyl CoA/acetyl are thought to exert effects on PDHa activity through changes in PDH-E1α phosphorylation [10, 24]. Because IL-6 injections did not affect PDH-E1α phosphorylation significantly neither in the fed nor the fasted state in the present study, changes in these parameters do not appear to be a likely mechanism for the observed IL-6-induced effects on PDH regulation.

It should be noted that IL-6 induced an increase in PDHa activity when PDHa activity was at a very low level due to the fasting conditions. This means that the PDHa activity after IL-6 injections in the fasted state still was much lower than the level of PDHa activity after IL-6 induced downregulation of PDH in the fed state. The mechanism behind such a switch is unknown and additional studies are needed to answer this. The observations that PDHa activity, PDH-E1α phosphorylation, PDK4 and PDP1 mRNA, as well as PDK4 protein were markedly changed by fasting in the present study are in accordance with previous studies [27, 28]. This shows that differences could be detected at the mRNA, protein phosphorylation, and activity level. Furthermore, the finding that fasting elicited 90 % reduction in PDHa activity in PBS, while IL-6 injection only resulted in a 30 % reduction demonstrates that the effect of 18 h of fasting is much more marked than a single IL-6 injection. This may indicate that IL-6 only contributes to the regulation of PDH for example by sensitizing PDHa to the metabolic needs of the cell. However, it may also be worth noting that the magnitude of reduction in PDHa activity in response to IL-6 injection is quite similar to the reduction previously reported in the exercise-induced increase in PDHa activity in human skeletal muscle, when muscle glycogen had been lowered prior to exercise [15]. This makes it possible that an enhanced IL-6 release contributed to the smaller exercise-induced increase in PDHa activity when muscle glycogen was reduced in the previous study [15]. Moreover, the similar magnitude of change in the present study and the previous human study indicates that the observed change in PDHa activity with IL-6 injections is similar to changes observed in a physiological setting in humans [15].

PDH-E1α phosphorylation is known to be the dominant regulatory mechanism determining PDHa activity. The unchanged PDH-E1α phosphorylation with IL-6 injection in the present study indicates that the observed changes in PDHa activity was not due to changes in phosphorylation level. Such discrepancy between PDHa activity and PDH-E1α phosphorylation has previously been reported in resting human and mouse skeletal muscle [14, 25], although the previous studies observed changes in PDH-1Eα phosphorylation without changes in PDHa activity. As effects of PDK and PDP expression and/or activity would be expected to influence the phosphorylation level of PDH-E1α, these PDH regulatory proteins do not seem to be in play in the IL-6-mediated effects. This suggestion is in line with the unaffected PDK4 and PDP1 protein level with a single IL-6 injection. Furthermore, the unchanged plasma glucose concentration and unchanged muscle glycogen in the fed state upon IL-6 injection (although an overall difference was observed for muscle glycogen) indicate that IL-6 did not change the carbohydrate availability in the fed state. Thus, it seems unlikely that changes in the metabolic state have contributed to the observed downregulation of PDHa activity. It may therefore be speculated that IL-6 has elicited alternative mechanisms like changes in acetylation state, because several acetylation sites have been identified on PDH [8].

Based on the previous findings that reduced muscle glycogen has been shown to enhance IL-6 transcription in human skeletal muscle [12] it may be expected that fasting would elevate muscle IL-6 mRNA levels. However, the finding that a fasting-induced elevation in IL-6 mRNA only was observed in IL-6 injected mice suggests that low glycogen is not sufficient to increase IL-6 mRNA, and that IL-6 exerts a positive feedback on the expression of IL-6 when muscle glycogen levels are low. In addition, the IL-6 induced intracellular signaling observed both in the fed and fasted state while IL-6 mRNA only increased significantly in the fasted state in the present study, may suggest that an IL-6-mediated effect on IL-6 expression requires a factor which is only available when muscle glycogen is low. As p38 has been implicated in regulating IL-6 expression [36] the observed fasting-induced increase in p38 protein content may have contributed to the elevated IL-6 mRNA. However, the observed reduction in p38 protein content with IL-6 injection does not support this possibility. Hence, the mechanism behind the fasting-induced increase in skeletal muscle IL-6 mRNA only when IL-6 was injected remains to be determined.

In conclusion, a single IL-6 injection reduces PDHa activity in mouse skeletal muscle in the fed state and increases PDHa activity in the fasted state. This suggests that IL-6-mediated PDH regulation contributes to regulating substrate choice, but that the metabolic state determines the outcome. IL-6 appears to regulate PDHa activity without clear changes in PDH-E1α phosphorylation and AMPK and p38 may be involved in the IL-6-mediated PDH regulation in the fasted state.
